# Transplantation of human neural stem cell prevents symptomatic motor behavior disability in a rat model of Parkinson’s disease

**DOI:** 10.1515/biol-2022-0834

**Published:** 2024-02-24

**Authors:** Fen Wang, Xiao-Yu Cheng, Yu-Ting Zhang, Qing-Ran Bai, Xiao-Qi Zhang, Xi-Cai Sun, Quan-Hong Ma, Xiong-Fei Zhao, Chun-Feng Liu

**Affiliations:** Department of Neurology and Clinical Research Center of Neurological Disease, The Second Affiliated Hospital of Soochow University, Suzhou 215004, China; Institute of Neuroscience, Soochow University, Suzhou, 215123, China; Key Laboratory of Spine and Spinal Cord Injury Repair and Regeneration of Ministry of Education, Orthopaedic Department of Tongji Hospital, School of Life Sciences and Technology, Tongji University, Shanghai, 200333, China; Shanghai Angecon Biotechnology Co., Ltd, Shanghai, 201318, China

**Keywords:** neural stem cell, motor behavior, dopamine, Parkinson’s disease, neurodegenerative disease, stem cell transplantation

## Abstract

Parkinson’s disease (PD) is a ubiquitous brain cell degeneration disease and presents a significant therapeutic challenge. By injecting 6-hydroxydopamine (6-OHDA) into the left medial forebrain bundle, rats were made to exhibit PD-like symptoms and treated by intranasal administration of a low-dose (2 × 10^5^) or high-dose (1 × 10^6^) human neural stem cells (hNSCs). Apomorphine-induced rotation test, stepping test, and open field test were implemented to evaluate the motor behavior and high-performance liquid chromatography was carried out to detect dopamine (DA), 3,4-dihydroxyphenylacetic acid (DOPAC), serotonin, and 5-hydroxyindole-3-acetic acid in the striatum of rats. Animals injected with 6-OHDA showed significant motor function deficits and damaged dopaminergic system compared to the control group, which can be restored by hNSCs treatment. Treatment with hNSCs significantly increased the tyrosine hydroxylase-immunoreactive cell count in the substantia nigra of PD animals. Moreover, the levels of neurotransmitters exhibited a significant decline in the striatum tissue of animals injected with 6-OHDA when compared to that of the control group. However, transplantation of hNSCs significantly elevated the concentration of DA and DOPAC in the injured side of the striatum. Our study offered experimental evidence to support prospects of hNSCs for clinical application as a cell-based therapy for PD.

## 1 Introduction

Parkinson’s disease (PD) is a chronic neurological disease caused by the decay or breakdown of the neurons in the brain that are responsible for producing dopamine [1,2]. PD exhibits a variety of clinical manifestations, encompassing both motor and non-motor symptoms, compared with other motor deficit diseases [3–7]. Neural stem cells (NSCs) have shown potential as a therapeutic option for PD due to their ability to replace damaged or lost neurons [8]. In 1992, Weiss and Reynolds [9] separated NSCs from the striatum of adult mice and first employed epidermal growth factor (EGF) to stimulate the growth and division of NSCs *in vitro*. Subsequently, studies have shown that the joint application of EGF and basic fibroblast growth factor (bFGF) can effectively promote the proliferation and self-renewal of NSCs, leading to the development of neurospheres derived from mammalian tissues [10,11]. However, to advance cell therapies, specifically those utilizing human NSCs (hNSCs), from the experimental stage to clinical application, it is necessary to establish stringent protocols for cell production and preservation following the regulations of good manufacturing practice (GMP).

To assess the feasibility of using hNSCs in a clinical study aiming at PD patients, we conducted a preclinical study to evaluate the efficacy of hNSCs in reducing neurological deficits in a rodent model of PD.

## 2 Methods and materials

### 2.1 Generation of hNSCs

hNSCs were derived from aborted fetal brain tissue (8–12 gestational week), which was obtained with full parental permission and approval from the Institutional Ethics Committee (2011 NO.103). Fetal midbrain tissue was separated and dissected at GMP facilities (Angecon Biotech, China), and then digested with papain (200 U/10 mL, Worthington, USA) for 30 min. The prepared single cell concentration was 1.0 × 10^5^ cells/mL and cultured with serum-free NSCs medium (Angecon Biotech, China), which consisted of a 1:1 mixture of Dulbecco’s modified Eagle’s medium and Ham’s F12 (Invitrogen), supplemented with B27 (2% v/v; Invitrogen), GlutaMAX (2% v/v; Invitrogen), MEM sodium pyruvate (2% v/v; Invitrogen), N2 formulation (1% v/v; Invitrogen), NAC (1.63 mg/mL; Sigma), bFGF (20 ng/mL; PEPROTECH), EGF (20 ng/mL; PEPROTECH), and LIF (10 ng/mL; PEPROTECH). The primary cells were grown for a period of 12–14 days in a humidified incubator at 37°C and 5% CO_2_. Then the neurospheres were dissociated with TrypLE^TM^ Express (Invitrogen, USA) to single cells, and seeded into the new T75 flasks at a concentration of 1 × 10^5^ cells/mL. Under the same culture conditions, hNSCs were further cultured and passaged, then frozen and stored in the three-level cell banks: the Seed Cell Bank (SCB, P3), the Master Cell Bank (MCB, P5), and the Working Cell Bank (WCB, P8).

Cell preparation: Cells in WCB were thawed and cultured for 14 days, then digested into single cells and washed with normal saline twice, then adjusted to the final concentrations of 2 × 10^5^ cells/10 µL and 1 × 10^6^ cells/10 µL, and packed.


**Informed consent:** Informed consent has been obtained from all individuals included in this study.
**Ethical approval:** The research related to human use has been complied with all the relevant national regulations, institutional policies and in accordance with the tenets of the Helsinki Declaration, and has been approved by the Institutional Ethics Committee (2011 NO.103). The research related to animal use has been complied with all the relevant national regulations and institutional policies for the care and use of animals, and has been approved by the Institutional Ethics Committee of the Second Affiliated Hospital of Soochow University (201511A113).

### 2.2 Identification of hNSCs

#### 2.2.1 Phenotype detection

The cell samples were subjected to centrifugation (500×*g*, 3 min), and the resulting supernatant was removed. Cells were fixed using a fixation/permeabilization kit (BD, USA) and then incubated at 4°C for 15 min. Next, 400 μL cleaning solution was added, and the cells were centrifuged again (500×*g*, 3 min). The cells were buffered in PBS for intracellular staining and incubated with Alexa Fluor® 647 Mouse Nestin (560393, BD) and Alexa Fluor® 488 Mouse anti-Sox2 (560301, BD), and then washed twice. Finally, 500 μL PBS was added to resuspend the cells, and detection was performed using flow cytometry (Cytoflex, Beckman) [12].

#### 2.2.2 Differentiation potential

The 12-well plates were coated with poly-l-lysine (P4707, Sigma, Germany) and hNSC neurospheres were inoculated together with the medium into the well-coated plates for adherent culture. After 5–8 h of culture, the neurospheres were fully adhered to the plates, and then the medium without cytokines was added in place of the original medium. After 2 weeks of culture, the cells were dispersed and adhered to the bottom, and spontaneously differentiated. The fixed differentiated cells were then exposed to a permeabilization mixture containing 10% normal donkey serum, 0.1% Triton, and PBS for 1 h. Then the cells were treated with primary antibodies (B-tubulin [T8860 sigma, Germany], GFAP [Z0334 DAKO, Denmark], and O4 [MAB345 Millipore, USA]) and incubated to allow for binding at 4°C overnight. After that, the cells were rinsed to remove any unbound primary antibodies and then incubated with secondary antibodies and DAPI. Images were taken using a fluorescence microscope (DMI 3000, Laica, Germany).

#### 2.2.3 Genetic test

Cells were lysed with Rneasy Mini Kit (Qiagen, Germany) and RNA was separated based on the manufacturer’s specified guidelines. RNA quantity was determined by Qubit^TM^ RNA HS Assay Kit using a Qubit Fluorometer (Life Technology, USA). RNAs were converted into cDNA using the Transcriptor First Strand cDNA Synthesis Kit (Roche, Switzerland). Light Cycler 480 SYBR Green I Master was used for qRT-PCR on a Light cycler 96 system (Roche, Switzerland). The specific primer sequences are outlined below: NCAM (Forward: GGCATTTACAAGTGTGTGGTTAC; Reverse: TTGGCGCATTCTTGAACATGA), NGFR (Forward: CCTACGGCTACTACCAGGATG; Reverse: CACACGGTGTTCTGCTTGT), Sox2 (Forward: TACAGCATGTCCT ACTCGCAG; Reverse: GAGGAAGAGGTAACCACAGGG), Pax6 (Forward: TGGGCAGGTATTACGAGACTG; Reverse: ACTCCCGCTTATACTGGGCTA), and GAPDH (Forward: GTGGACCTGACCTGCCGTCT; Reverse: GGAGGAGTGGGTGTCGCTGT).

#### 2.2.4 Karyotype test

Standard G-Band analysis was used for sample processing, and BX53 microscope (Olympus) was used to take photos. Chromosome numbers were calculated, and its characteristics were observed through images, and chromosome karyotype matching was carried out. At least 20 cell groups were selected from each sample for observation and counting to check whether there were abnormal conditions such as chromosome deletion and ectopic.

### 2.3 Animals

The animal protocol was approved by the Institutional Ethics Committee of the Second Affiliated Hospital of Soochow University (201511A113). Adult Sprague Dawley rats of both genders (180 ± 10 g) were sourced from Shanghai SLAC Laboratory Animal Co., Ltd (Shanghai, China). The rats were accommodated (five rats per cage) in a temperature-controlled room (22–25°C) with 12 h light/dark cycle. Food and water were offered to the rats without limitation. All animal procedures adhered to the established guidelines of the Animal Use and Care Committee of Soochow University. Fifty-six rats were used to build PD models by 6-hydroxydopamine (6-OHDA, H116, Sigma, USA) injection and 42 rats were successfully modeled. The rats were allocated to four groups at random: (1) sham group (*n* = 10), (2) PD model group (6-OHDA, *n* = 14, male, 7; female, 7), (3) low-dose hNSCs group (2 × 10^5^ hNSCs, *n* = 9, male, 4; female, 5), and (4) high-dose hNSCs group (1 × 10^6^ hNSCs, *n* = 19, male, 9; female, 10). All rats in (2), (3), and (4) groups were given saline or hNSCs suspension intranasally once a week for 4 weeks. After the first hNSCs/saline treatment, apomorphine (APO)-induced rotation test, stepping test, and open field test were performed every 2 weeks for 12 weeks.


**Ethical approval:** The research related to animal use has been complied with all the relevant national regulations and institutional policies for the care and use of animals, and was approved by the Institutional Ethics Committee of the Second Affiliated Hospital of SoochowUniversity (201511A113).

### 2.4 Establishment of 6-OHDA-induced PD rat model

The rats were administered 3% isoflurane until the loss of righting reflex was observed, following which anesthesia was maintained using 1.5% isoflurane, and the rats were secured in a stoelting stereotaxic apparatus (RWD Co., Shenzhen, China). The rat brain atlas was consulted to determine the location [13]. 6-OHDA in saline (16 μg/8 μL) was administered via injection into the left medial forebrain bundle (MFB) through a 10 μL Hamilton syringe at a speed of 0.5 μL/min. The left MFB’s coordinates (two spots) were as follows: (1) −1.8 mm anteroposterior, −2.5 mm mediolateral, and −7.5 mm dorsoventral; (2) −1.8 mm anteroposterior, −2.5 mm mediolateral, and −8.0 mm dorsoventral (from the dura) from the bregma. The syringe was slowly extracted from the MFB after remaining in place for a duration of 4 min. Following a 3-week healing period after the surgery, apomorphine hydrochloride (APO, 013-18313, Wako, Japan)-induced rotation tests were performed. Only rats that displayed a rotational frequency above 210 turns per 30 min during rotation tests were deemed eligible for inclusion in the PD model [14].

### 2.5 Intranasal administration of hNSCs

Rats were anesthetized via inhalation of isoflurane gas delivered in a flow of oxygen, with a concentration of 3.0% for induction and 1.5% for maintenance. The rats were then positioned in the supine position. Each rat was given 10 μL (5 μL/side) of hNSCs cell suspension or saline bilaterally. A thin hose of 0.5–0.7 cm in length was placed at the needle of the microsyringe. The cells suspension was absorbed and extended into the left nasal cavity, and slowly injected into the nasal cavity and maintained its posture for 5 min. After 15 min, the cell suspension was given to the right nostril in the same way. After administration, the rats were kept in supine position for 5 min. Anesthesia was discontinued and the rats were returned to their cages when aroused [12,15].

### 2.6 Behavioral experiments

#### 2.6.1 APO-induced rotation test

Using a video camera, the contralateral rotations caused by APO (0.25 mg/kg, subcutaneous) were measured and documented for a period of 30 min during each recording session [16].

#### 2.6.2 Stepping test

While securing the hindlimbs of the rat with one hand, the experimenter ensured that the forelimbs of rats were unconstrained but kept in touch with the table. The rat was repositioned to the side (90 cm in 5 s) in the forehand direction, and the frequency of steps to both forelimbs was counted and documented [17].

#### 2.6.3 Open field test

The rat was placed in the middle of a cubic container (80 × 80 × 40 cm^3^) that was equipped with a camera. The behavioral box had a black-colored interior and bottom surface, which contrasted with the rats’ coloring. Using a Flex field/Open Field Activity System (ANY-maze; Stoelting, Wood Dale, IL, USA), the locomotor tracks of the rats were continually recorded. The observations of each rat were conducted and recorded for 300 s, and the process was repeated three times at 10 min intervals.

### 2.7 Immunostaining of tyrosine hydroxylase (TH)

To determine the viability of the TH neurons in the substantia nigra (SN), we performed immunostaining as described previously [18].

### 2.8 High-performance liquid chromatography (HPLC)

At the 12th week, after behavioral determination, dopamine (DA), 3,4-dihydroxyphenylacetic acid (DOPAC), 5-hydroxyindole-3-acetic acid (5-HIAA), and serotonin (5-HT) in the striatum were detected by HPLC with an electrochemical detector (Antec, Zoeterwoude, Netherlands). The striatal tissues were lysed and centrifuged at 12,000 rpm for 20 min at 4°C in 4% perchloric acid. The supernatants were filtered through a 0.22 μm syringe filter before applying to the HPLC system. A mobile phase contained NaH_2_PO_4_ 100 mM, sodium octanesulfonate 0.74 mM, EDTA-Na_2_ 0.027 mM, KCl 2 mM, and 15% (vol/vol) methanol. A C18 chromatographic column (2.1 mm, 100 mm, 3 mm, Antec) was used to analyze the qualitative and quantitative content of neurotransmitter. The standard substances (DA, H8502; DOPAC, 11569; 5-HT, H7877; 5-HIAA, H8876, purchased from Sigma) were used for quantitative analysis, and the neurotransmitter was analyzed at a speed of 0.20 mL/min by the HPLC pump.

### 2.9 Statistical analysis

Data gathered from behavioral tests and HPLC were checked for normality of distribution and homogeneity of variance and displayed as mean ± SEM. Data of behavioral tests were analyzed using the repeated measurement ANOVA followed by multiple comparisons performed by SPSS 22.0 statistical software. Data of HPLC were analyzed with Kruskal–Wallis test and Dunnett non-parametric test for further multiple comparison test. Differences with *p*-values less than 0.05 (two sided) were deemed to be statistically significant.

## 3 Results

### 3.1 Characteristics of hNSCs

**Figure 1:**
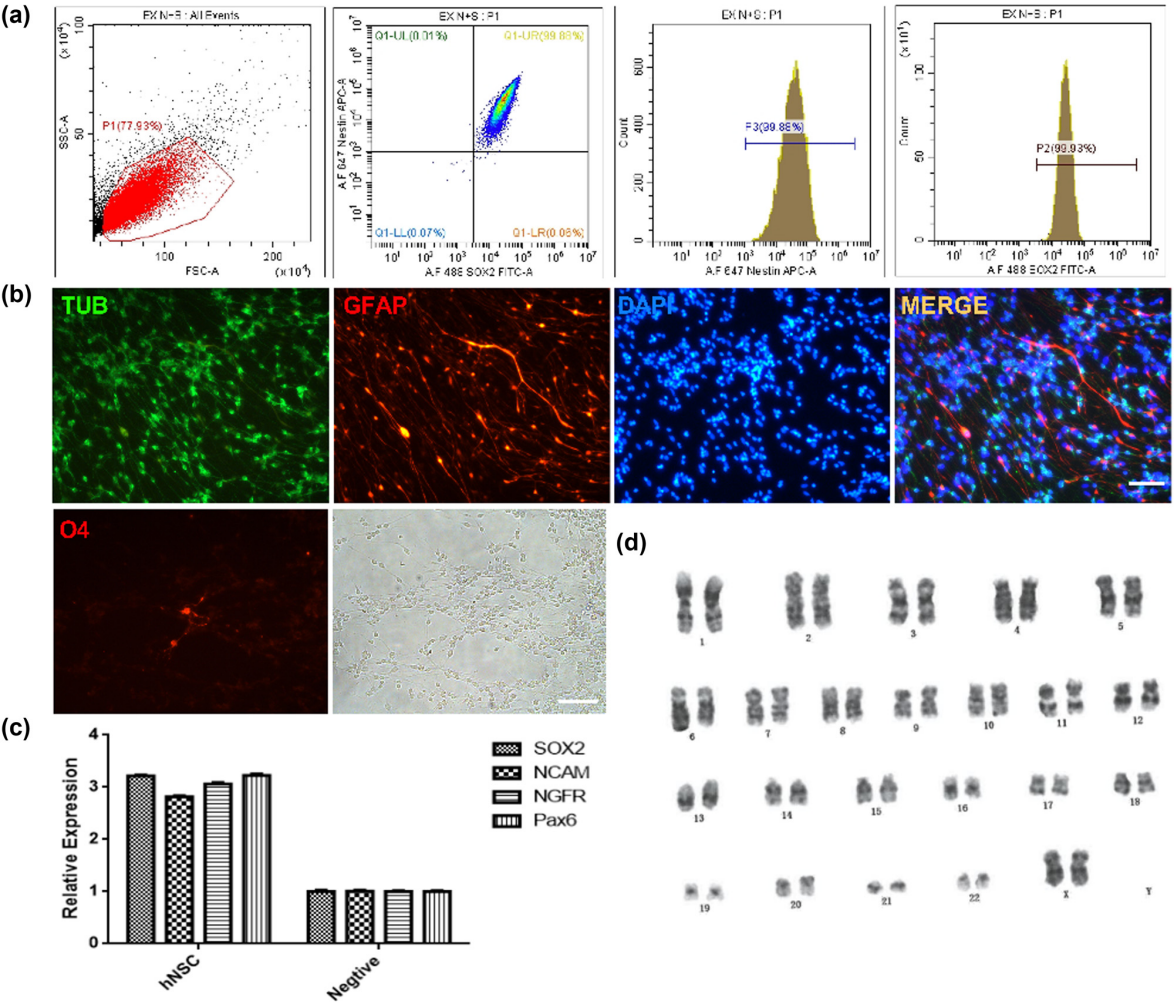
Characteristics of hNSCs: (a) phenotype detection, (b) differentiation potential, (c) specific gene expression, and (d) karyotype test. Scale bar: 100 μm, TUB: B-tubulin; GFAP: glial fibrillary acidic protein.

Flow cytometry was used to monitor the phenotype of NSCs. The expressions of Nestin and SOX2 were higher than 90% (Figure 1a). Fluorescence photomicrograph showed that NSCs could different into neurons (B-tubulin positive), astrocyte (GFAP positive), and oligogendrocyte (O4 positive) (Figure 1b). qRT-PCR indicated that the expressions of specific genes of NSCs (SOX2, NCAM, NGFR, and Pax6) were significantly higher than that of peripheral blood mononuclear cells (Figure 1c). The karyotype of the cell line was normal (46, XX) (Figure 1d). As a summary, these results signify that the quality of NSCs meets the requirements.

### 3.2 APO-induced rotation test

**Figure 2:**
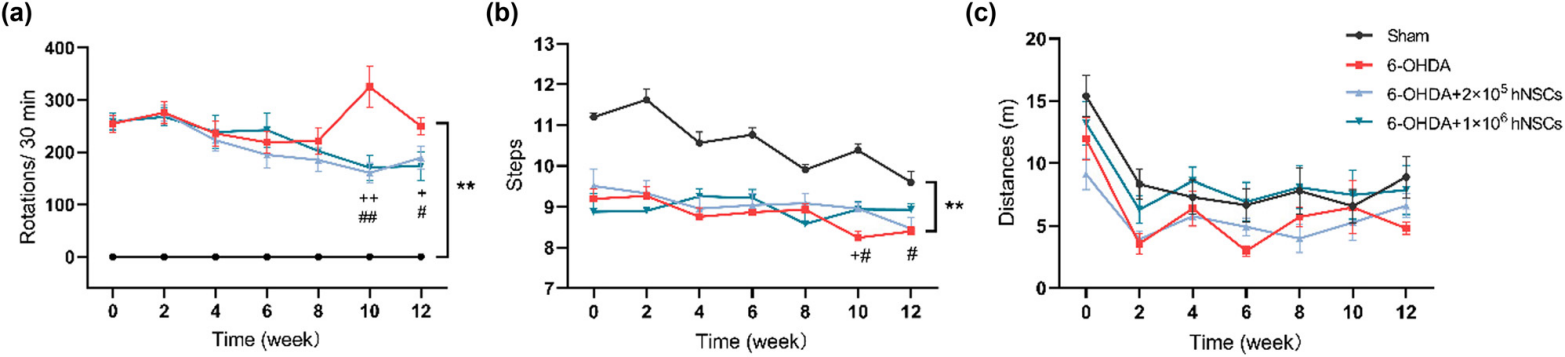
Behavior test of rats in all groups: (a) APO rotation test, (b) stepping test, and (c) open field test. **: *p* < 0.01, 6-OHDA group vs sham group; +: *p* < 0.05, 6-OHDA + 2 × 10^5^ hNSCs group vs 6-OHDA group; ++: *p* < 0.01, 6-OHDA + 2 × 10^5^ hNSCs group vs 6-OHDA group; #: *p* < 0.05, 6-OHDA + 1 × 10^6^ hNSCs group vs 6-OHDA group; ##: *p* < 0.01, 6-OHDA + 1 × 10^6^ hNSCs group vs 6-OHDA group.

After the 6-OHDA injection, the statistical analysis demonstrated a significant difference in the count of rotations induced by APO between the animals that received the injections and the sham group (Figure 2a). In addition, we observed that the 6-OHDA injections resulted in alterations in the motor function of the animals. At the 10th and 12th week, the number of APO-induced rotation in both 2 × 10^5^ and 1 × 10^6^ hNSCs group was significantly lower than that in 6-OHDA model group, respectively (*p* < 0.01 and *p* < 0.05) (Figure 2a). The rotations did not exhibit any significant differences between low-dose and high-dose hNSCs treatment group.

### 3.3 Stepping test

There was a statistically significant difference observed in the number of steps between the PD model group and the sham group, with the former showing a lower count. At the 10th week, the number of steps observed in the 2 × 10^5^ hNSCs treatment group was significantly greater than that in the 6-OHDA model group (*p* < 0.05). Moreover, there were also significant differences between 1 × 10^6^ hNSCs treatment group and 6-OHDA model group at the 10th and 12th week after hNSCs administration (*p* < 0.05). At other time points, the differences were not significant (Figure 2b).

### 3.4 Open field test

Rats were filmed while freely moving in a square flat arena for 300 s and the path followed by the animals was tracked and analyzed. The total traveled distance of rats in the 6-OHDA group showed a trend of reduction compared to the sham group, but the difference did not reach statistical significance. The results did not show any significant differences in the traveled distance between hNSCs treatment group and 6-OHDA model group at all time points (Figure 2c).

### 3.5 Immunostaining of TH in the SN

**Figure 3:**
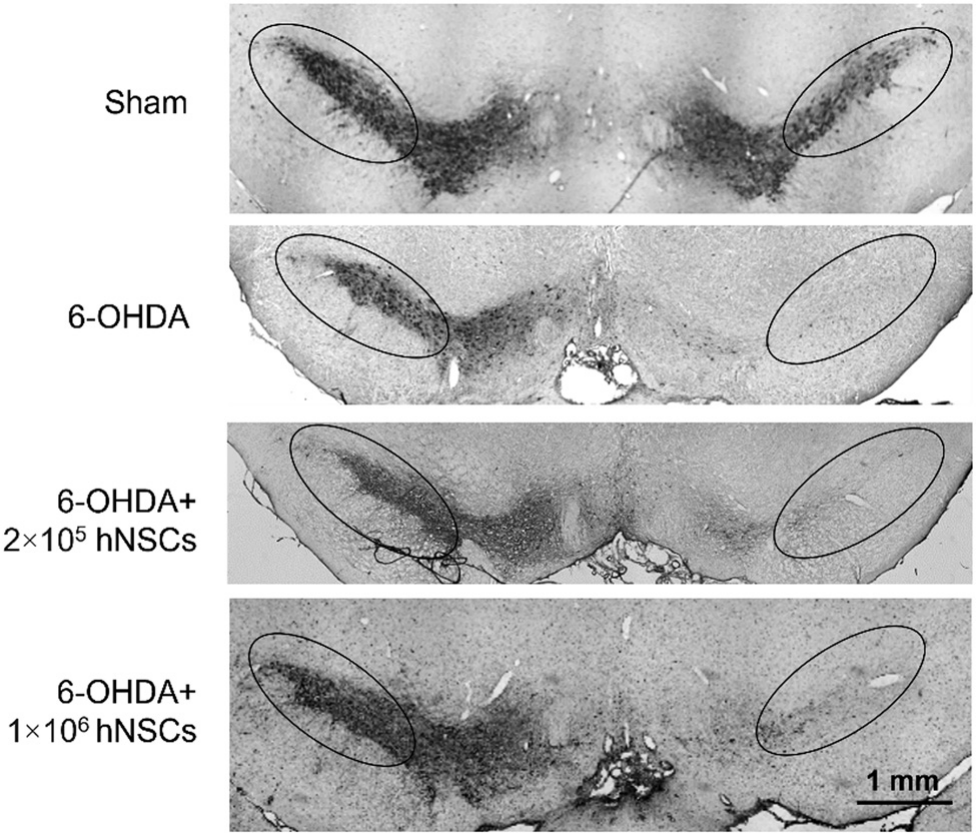
Representative images of TH immunoreactivity in the SN from rats. The right side of each panel shows the injected effect of 6-OHDA. TH signal was not significantly different between the 6-OHDA + 2 × 10^5^ hNSCs group and the 6-OHDA group (7.19 ± 1.35 vs 4.94 ± 0.67, *p* = 0.15), but was significantly higher in the 6-OHDA + 1 × 10^6^ hNSCs group compared to that of the 6-OHDA group (25.19 ± 8.02 vs 4.94 ± 0.67, *p* < 0.05). *n* ≥ 3 in each group.

Following the motor function test, we conducted immunohistochemical analysis to investigate the recovery of dopaminergic neurons in the SN using the TH antibody. The 6-OHDA injected animals showed significantly reduced TH intensity both in the injured and contralateral side of SN in comparison to the control rats. However, TH intensity was restored in the PD rats treated with 1 × 10^6^ hNSCs (Figure 3).

### 3.6 Levels of DA, DOPAC, 5-HIAA, and 5-HT in the striatum

**Figure 4:**
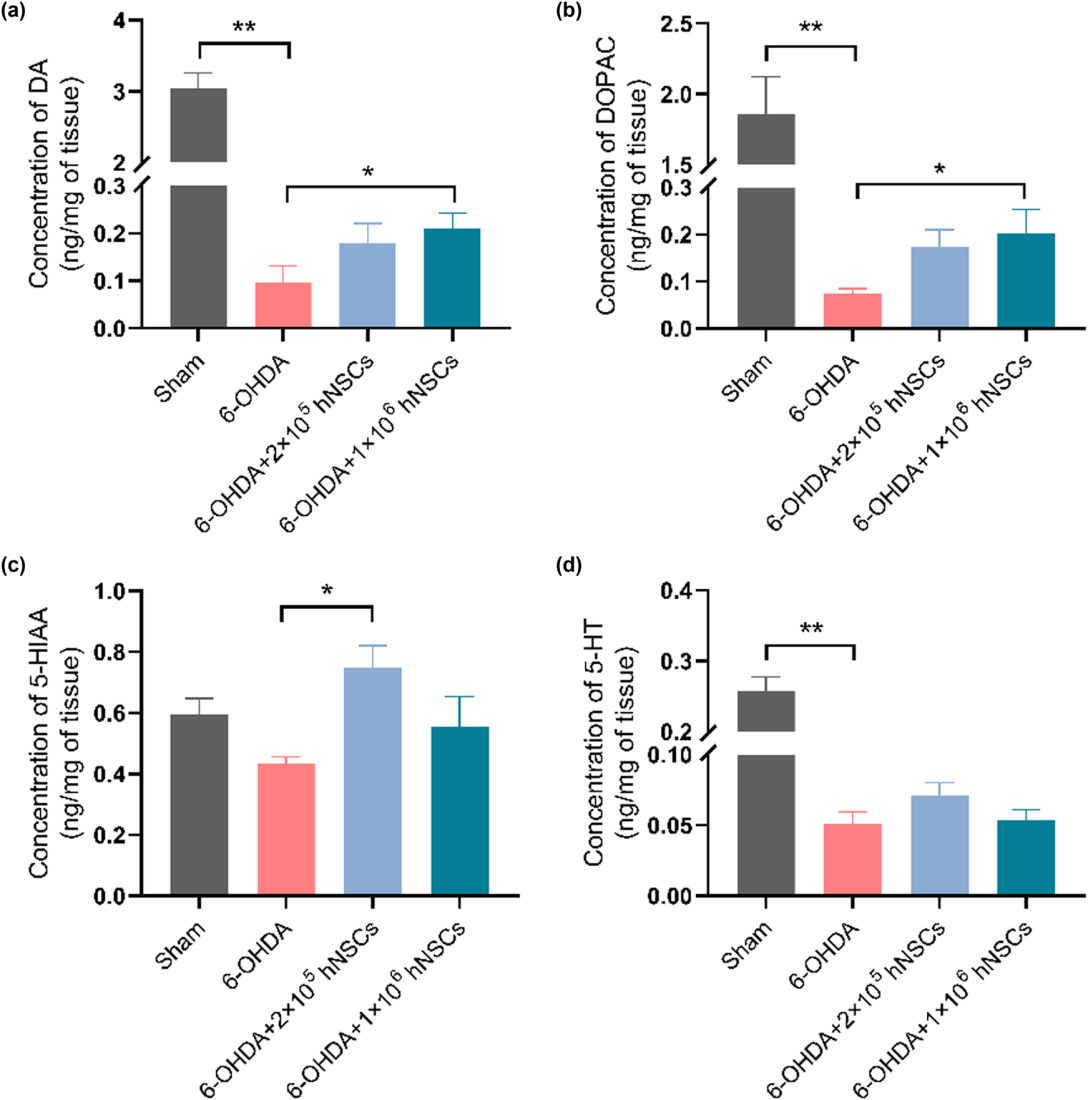
Concentration of DA (a), DOPAC (b), 5-HIAA (c), and 5-HT (d) in the injured side of striatum. 6-OHDA: 6-hydroxydopamine; hNSCs: human neural stem cells; DA: dopamine; DOPAC: dihydroxyphenyl acetic acid; 5-HT: serotonin; 5-HIAA: 5-hydroxyindole-3-acetic acid. *: *p* < 0.05; **: *p* < 0.01.

HPLC was utilized to measure the contents of DA, DOPAC, 5-HIAA, and 5-HT in the injured side of striatum at the 12th week in all groups. As shown in Figure 4, the contents of DA, DOPAC, and 5-HT in the injured side of striatum in the 6-OHDA group exhibited a statistically significant reduction contrasted with the sham group (*p* < 0.01). The content of 5-HIAA showed a decreased trend but the difference did not meet the threshold for statistical significance. After hNSCs administration, the content of DA and DOPAC in the injured side were significantly increased in 1 × 10^6^ hNSCs group, respectively, compared to those of the 6-OHDA group (*p* < 0.05). The content of 5-HIAA was significantly increased in the 2 × 10^5^ hNSCs group, compared to that of the 6-OHDA group (*p* < 0.05).

## 4 Discussion

Recent studies have confirmed the feasibility and safety of employing NSCs clinically to manage various neurodegenerative conditions, including amyotrophic lateral sclerosis [19,20], spinal cord injury [21], Alzheimer’s disease [22,23], and Huntington’s disease [24,25]. Some studies have offered evidence to support the idea that the replacement of cells could have the ability to reverse the neurobehavioral symptoms in certain patients with neurodegenerative diseases [6,10,26,27]

In order to establish a stringent protocol before the clinical application of hNSCs, a clearly defined animal model of PD was employed in this study. The results of the APO-induced rotation test (Figure 2a) demonstrated that the rats injected with 6-OHDA had a pronounced turning behavior, indicating a significant impairment in the proper functioning of the brain’s dopamine-producing system in contrast to that of the sham group. Furthermore, the animals’ movement was also found to be impaired, as determined by the stepping test. As evaluated through neurobehavioral tests, significant enhancements in motor performance were observed in the PD animals following hNSCs treatment in comparison to the untreated PD group (Figure 2). In addition, treatment with hNSCs significantly increased the quantity of cells expressing TH in the SN of PD animals. Moreover, we found animals receiving 6-OHDA injection showed a significant reduction in the levels of DA, DOPAC, 5-HIAA, and 5-HT in the injured side of striatum when contrasted with sham group. However, administration of hNSCs led to a significant increase in levels of the neurotransmitter dopamine and its metabolite DOPAC in the injured side of the striatum (Figures 3 and 4). These results in the present study indicated that hNSCs significantly improve the motor behavior and increase the striatal dopamine in the rat model of PD.

There have been several preclinical studies that have demonstrated the potential of NSCs as a therapeutic option for PD [28–34]. The level of DA transporter immunoreactivity in the host brain tissue was found to be positively linked to the degree of functional recovery observed [35]. Simultaneous transplantation of NSCs overexpressing glial cell line-derived neurotrophic factor and fetal dopaminergic neurons was found to mitigate motor symptoms in a rat model of PD in one study [28]. The secretome of human NSCs was characterized in another study, which revealed that its application partially modulated DA neurons cell survival and improved the motor deficits of PD animals [30]. Qian et al. observed an increase in the suitability of host brain environments following the co-transplantation of NSCs overexpressing Nurr1 and microglia [32]. Using a high-throughput quantitative proteomic approach, Zuo et al. characterized the protein profiles of brain regions associated with PD in 6-OHDA-injected Parkinsonian mice, including the striatum, SN, olfactory bulb, and subventricular zone [33]. Human NSC-derived extracellular vesicles exhibited neuroprotective properties by decreasing reactive oxygen species and pro-inflammatory cytokines in an *in vitro* transwell system and a PD model [36]. Consistent with these findings, our current study validated that hNSCs significantly improved motor behavior and increased striatal dopamine levels in PD rats. Additionally, we evaluated the efficiency of varying doses of hNSCs and found that the impact of hNSCs on motor function was dependent on the dosage.

The underlying mechanisms of the hNSCs’ effects on PD are not fully understood. By replacing the lost dopaminergic neurons in the SN, hNSCs may restore the production of dopamine and improve the symptoms of PD [37–42]. hNSCs may also secrete neurotrophic factors, which are proteins that support the survival and expansion of neurons [43–45]. Neurotrophic factors may help protect and promote the growth of dopaminergic neurons in the SN, potentially slowing or halting the advancement of PD [33]. In addition, hNSCs may have immunomodulatory effects, meaning they can regulate the immune response in the brain [43]. PD is associated with inflammation in the brain, and hNSCs may be able to reduce this inflammation, leading to a reduction in the symptoms of the disease [46]. Finally, hNSCs may also have a neuroprotective effect. They may be able to protect existing neurons from further damage, potentially slowing the progression of the disease [30,47]. Human embryonic stem cell transplantation clinical trials for PD treatment are presently ongoing in Australia < NCT02452723 > [48] and China < NCT03119636 (Chinese ASZQ-003) > [49], and other trials are set to commence soon [50,51].

## 5 Conclusions

In conclusion, transplantation of hNSCs improves symptomatic motor behavior, prevents symptom progress, and increases striatal dopamine in a rat model of PD, which indicates that hNSCs have the possibility of being a preventive and therapeutic method for PD. Our study offers experimental evidence for the potential use of hNSCs in cell-based therapy for PD in clinical settings.
